# Enhancement in statistical and image analysis for *in situ* µSXRF studies of elemental distribution and co-localization, using *Dioscorea balcanica*

**DOI:** 10.1107/S0909049512050170

**Published:** 2013-01-29

**Authors:** Tanja Dučić, Manuela Borchert, Aleksandar Savić, Aleksandar Kalauzi, Aleksandra Mitrović, Ksenija Radotić

**Affiliations:** a DESY, Notkestrasse 85, D-22607 Hamburg, Germany; b Institute for Multidisciplinary Research, University of Belgrade, Kneza Višeslava 1, 11000 Belgrade, Serbia

**Keywords:** X-ray fluorescence microscopy, image analysis, statistical analysis, *Dioscorea balcanica* Košanin

## Abstract

Synchrotron-based X-ray microfluorescence (µSXRF) is an analytical method suitable for *in situ* investigation of the distribution of micronutrient and macronutrient elements in several-micrometres-thick unstained biological samples, *e.g.* single cells and tissues. Elements are mapped and quantified at sub-p.p.m. concentrations. In this study the quantity, distribution and grouping/co-localization of various elements have been identified in straight and twisted internodes of the stems of the monocotyledonous climber *D. balcanica* Košanin. Three different statistical methods were employed to analyse the macro­nutrient and micronutrient distributions and co-localization. Macronutrient elements (K, P, Ca, Cl) are distributed homogeneously in both straight and twisted internodes. Micronutrient elements are mostly grouped in the vasculature and in the sclerenchyma cell layer. In addition, co-localization of micronutrient elements is much more prominent in twisted than in straight internodes. These image analyses and statistical methods provided very similar outcomes and could be applied to various types of biological samples imaged by µSXRF.

## Abstract 2

Synchrotron-radiation-based X-ray microfluorescence has been used for *in situ* investigation of the distribution of micronutrient and macronutrient elements in an unstained cross section of a stem of monocotyledonous liana plant *Dioscorea balcanica* Košanin. The elemental allocation has been quantified and the grouping/co-localization in straight and twisted stem internodes has been analysed.

## 1. Introduction

Synchrotron-based X-ray microfluorescence (µSXRF) microscopy is well suited for *in situ* investigations of elemental distribution in whole or thick-sectioned unstained biological samples such as single cells or tissues, with a sub-p.p.m. detection limit (Paunesku *et al.*, 2009 ▶). Using X-ray microfluorescence, data on multiple elements can be collected simultaneously without sample destruction. The high penetration capability of X-rays combined with a high spatial resolution makes X-ray fluorescence an ideal tool for two- and three-dimensional structural visualization at the subcellular level (reviewed by Ice *et al.*, 2011 ▶). X-ray fluorescence is particularly suited for quantifying trace elements in cells without the need for artificial dyes or fluorophores. In plant science, µSXRF may be used for obtaining spatially resolved data on the element distribution in tissues. This method may forward our knowledge of the changes in elemental distribution in plants grown under various external conditions (Punshon *et al.*, 2009 ▶). However, the overall number of studies applying µSXRF analysis is still small in plant science (Lombi *et al.*, 2011 ▶).

In this study we used statistical/image analysis of µSXRF data to describe elemental associations in *Dioscorea balcanica* Košanin stem sections as a model system.

*Dioscorea balcanica* (Dioscoreaceae) is an endemic species, a tertiary relict of the Balkan peninsula, and only wild *Dioscorea* species currently thrive in this area. This plant is a herbaceous monocotyledonous dioecious tuberous perennial liana, growing to a length of 2 m with a climbing stem. Many wild *Dioscorea* species are a very important source of secondary metabolites (diosgenin and related steroidal saponins) used in the pharmaceutical industry and medicine (Furmanowa & Guzewska, 1988 ▶; Šavikin-Fodulović *et al.*, 1998 ▶). Monocotyledons lianas offer an interesting comparison with dicotyledonous lianas in terms of functional anatomy (Carlquist, 1991 ▶). The scattered bundles typical of monocotyledonous stems resemble the cable-like construction of many lianas, with vascular strands included in a soft parenchyma (Isnard *et al.*, 2009 ▶).

In this study we compare the quantity and distribution of different elements in twisted and straight internodes of this liana species, and analyse elemental co-localization in these two types of internodes. Several image analysis techniques and statistical methods were applied: principal component analysis, image multiplication and probability density estimation. These data contribute new insight to research on mechanisms and properties of the twining machinery of climbing plants.

## 2. Experiment

### 2.1. Plant growth and sample preparation

*Dioscorea balcanica* plants were grown from tubers in a mixture of peat and perlite (1:1) in a greenhouse in Belgrade (44° 49’ N, 20° 29’ E), under natural day length, from April (14 h/10 h photoperiod) to June (15 h/9 h photoperiod), and at a temperature of 298 K. We used vegetative plants of length ∼1.2 m with 10–12 internodes fully elongated (the last counted internode is the one still circumnutating). Plants with twined and/or straight sixth and seventh internodes (about 50–30 cm, respectively, from the apex, *i.e.* four to five internodes below the internode still circumnutating) were selected for analysis. Sections of stem tissue of the first internode (which is always straight), sixth and seventh internodes (either straight or twisted) were prepared by free-hand sectioning with a razor blade and subsequently freeze dried. The samples’ thickness was obtained using a confocal laser scanning microscope (Zeiss LSM 510 META) and calculated using an LSM image browser. Visible-light fluorescence microscopy was carried out using a Nikon Eclipse T*i* microscope (NIKON GMBH).

### 2.2. X-ray fluorescence microscopy

X-ray fluorescence is generated by the interaction of X-rays with matter. If the incident X-ray energy is equal (or higher) to the binding energy of a core electron (*K*, *L* or *M* shell), the electron is ejected to the continuum (photoelectric effect). The principle and applications of X-ray fluorescence microscopy are explained in the review of Sakdinawat & Attwood (2010 ▶). Each element has a unique fluorescence spectrum, so this technique allows multi-element analysis. X-ray fluorescence provides qualitative and quantitative elemental distribution information, and in combination with X-ray spectroscopy it is the only known technique which can determine oxidation states of elements *in situ* (Ice *et al.*, 2011 ▶). X-ray fluorescence analysis of small biological samples demands a high photon flux and a small X-ray beam spot size in order to obtain two-dimensional elemental maps with a good spatial resolution and sufficient lower limits of detection. Furthermore, fast scanning is required to avoid radiation damage of samples.

The experiment was performed at beamline L at DORIS III (DESY, Hamburg, Germany), a beamline dedicated to micro-analyses of samples by X-ray fluorescence, X-ray spectroscopy (XAFS) and X-ray diffraction at energies between 5 and 40 keV. The experimental set-up included a multilayer monochromator for high photon flux and a polycapillary half lens to focus the incoming X-ray beam to a spot of diameter 15 µm. A silicon drift detector positioned at 90° to the incoming X-ray beam equipped with a standard collimator with a 2 mm pinhole was used to collect the fluorescence signal. The excitation energy was set to 13 keV in order to enable simultaneous detection of Fe, Zn, Cu, Ni and Mn (micronutrient elements) as well as Ca, K, S and Cl (macronutrient elements). A 100 µm-thick NIST standard (SRM 612) measured with the same set-up was used for signal calibration in order to allow quantification of element concentrations in the samples (Hinton, 2007 ▶). Two-dimensional maps were created by scanning the sample through the X-ray beam. At each point a µSXRF spectrum was collected. The spectra were subsequently fitted using *AXIL* software (Vekemans *et al.*, 1995 ▶, 2004 ▶). This software allows interactive as well as batch processing of large data sets and it is particularly well suited for data obtained by X-ray imaging. The algorithms employed are described in detail elsewhere (Vekemans *et al.*, 1995 ▶, 2004 ▶; Solé *et al.*, 2007 ▶).

### 2.3. X-ray image analysis

#### 2.3.1. Pre-processing

The first step in the image analysis was to define regions of interest in order to avoid areas of background noise and calculate the actual area of each sample so that element concentrations could be normalized.

Since not all of the elements were present in each image, it was necessary to construct logical true–false matrices for the presence of each element by measuring the level of background noise and setting an appropriate threshold level. The final logical matrix which defines the specimen was calculated by using logical ‘or’,
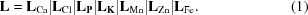
Further operations were performed on modified raw images (matrix) according to the following formula,
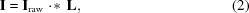
where **I** is the analyzed image, **I**_raw_ is the starting raw image, **L** is the logical true–false matrix which defines the object extracted from the background, and 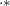 denotes element-by-element multiplication of matrices, meaning that only elemental intensities of pixels residing on the same *x* and *y* positions were multiplied.

Calculations were performed by using software package *Matlab-2007*.

#### 2.3.2. Principal component analysis

Principal component analysis (PCA) is a widely used analytical technique that allows one to reduce the number of variables and to identify relationships between variables which allows their classification. Orthogonal transformation was performed to convert sets of correlated observation (*i.e.* single-element distribution images) into a set of linearly uncorrelated variables (principal components). PCA was performed using the software *STATISTICA8*.

#### 2.3.3. Image multiplication

In order to examine element collocation in the sections of stems of *Dioscorea balcanica*, images were multiplied element-wise. Whenever certain pairs of elements showed a similar distribution pattern, regions with higher concentration could be easily identified due to significantly enhanced contrast compared with single-element distribution maps. Otherwise, if the metals were not co-local­ized in the same areas, image multiplication produced images without marked ‘enhanced contrast features’. Two element groups have been identified based on factor scores from PCA analysis. Furthermore, logarithms of products and their absolute values were calculated in order to perform colour rendering without loss of information. More precisely, if a linear gradient was to be applied for image colour rendering and the colour linearly corresponded to product values, significant areas of the resulting ‘product’ image would become excessively dark and featureless. A logarithm function was applied because it enhanced features in the dark region of the ‘product’ image (corresponding to the area of elemental co-localization), while suppressing them in the bright domain (corresponding to the area of independent distribution of two elements under investigation). Calculations were performed according to the equations
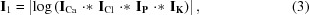


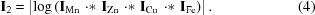
Image multiplication was computed by using the software package *Matlab-2007*.

#### 2.3.4. Probability density estimate

Probability density estimate (PDE) provides important information about element concentrations of the images. As PDE analysis requires vectors as input data, images were converted into vectors by using the following reshaping algorithm,
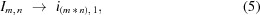
where *m* is the number of pixels in each column, *n* is the number of pixels in each row and 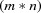 is the product of the number of column and row pixels used for converting the matrix to vector.

PDE data are presented as normalized histograms that take into account the analysis of element distribution in the whole image. The combination of PDE with image multiplication and PCA enabled us to make conclusions about the distribution of each element and learn whether its distribution pattern was unique or whether it co-localized with other elements.

PDE was calculated by using software package *Matlab-2007*.

## 3. Results and discussion

**Figure 1:**
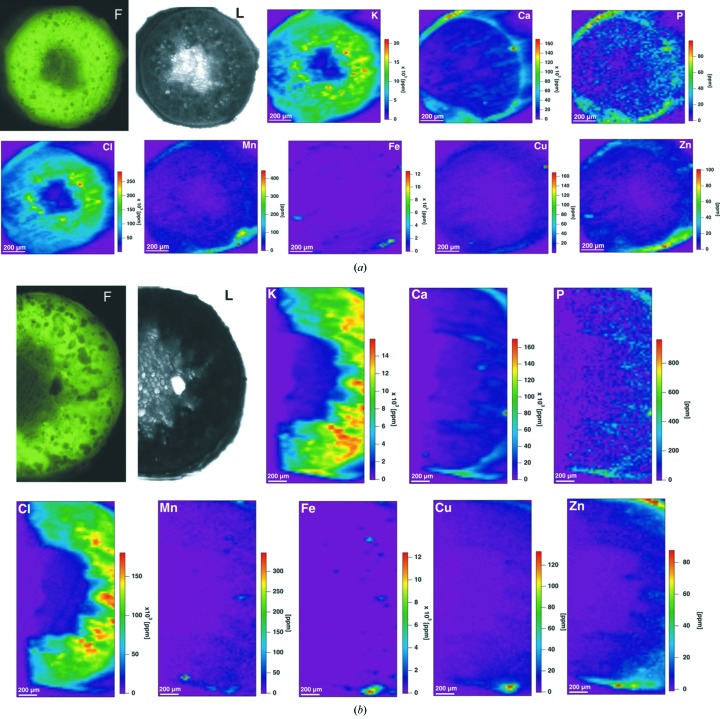
X-ray fluorescence microscopy images of sections of the *Dioscorea balcanica* sixth internode, (*a*) twisted and (*b*) straight, at energy 13 keV. F: autofluorescence of the stem cross section; L: light microscopy of the investigated area. X-ray scanning mapping of K, Ca, P, Cl, Mn, Fe, Cu and Zn are shown. Scan areas: 1060 × 1450 µm (*a*) and 1320 × 1370 µm (*b*); pixel size: 15 µm; dwell time: 1 s.

By using optical light fluorescence microscopy on the cross sections of the sixth internodes of *D. balcanica* stems either twisted [*F* and *L* in Fig. 1(*a*) ▶] or straight [*F* and *L* in Fig. 1(*b*) ▶], it was possible to distinguish (i) an epidermal cells layer, (ii) a layer of sclerenchymal cells, (iii) a zone of vascular bundles scattered through parenchyma cells, and (iv) thin-walled hexagonal parenchyma cells forming pith in the centre of the stem. X-ray fluorescence images of the same samples with different element distributions and concentrations in *D. balcanica* sections of the sixth internodes are shown in Fig. 1 ▶ together with light micrographs [(*a*) twisted and (*b*) straight stem sections]. The scale bar shows elemental concentrations in p.p.m. Background was removed according to equation (2). The imaged areas were chosen to be approximately similar in size. The pixel size (15 µm) and the dwell time (1 s) were the same for all measurements. Owing to the confining beam time at the synchrotron light source as well as a long exposure time per pixel, only a small number of samples and small sample size can be analysed within the experimental time. Concerning the experimental conditions and plant individuality, the whole cross section of the twisted internodes and half stem tissue of the straight internodes were analysed. Due to this limitation we did not investigate the botanical aspect of these physiological appearances, but we used this plant as a model system for the statistical aspects of analyses.

The highest concentration was observed for K, Ca, Cl and Fe in both straight and twisted internodes (Fig. 1 ▶). Apparently the co-localization grouping of elements is more expressed in the twisted internodes (Fig. 1*a* ▶) than in the straight ones (Fig. 1*b* ▶).

**Figure 2:**
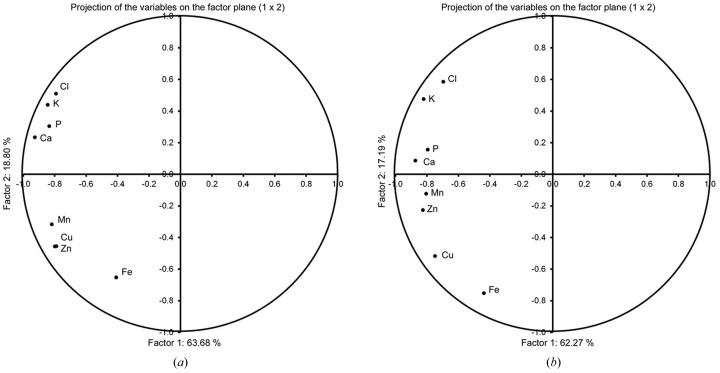
Principal component analysis plots. The images from Fig. 1 ▶ were used as the input data. The first and second principal components are presented, (*a*) for the twisted internode and (*b*) for the straight internode. Grouping of elements was based on second principal component values.

The patterns of all element distributions were analysed using three analytical statistical methods. Fig. 2 ▶ shows results of PCA for the images shown in Fig. 1 ▶ for the sixth twisted (*a*) and straight (*b*) internodes. The values of factor scores indicate both similarities and differences in distribution of elements in the corresponding sections. It was observed that in the twisted sixth internode elements were grouped into two groups (Fig. 2*a* ▶). Four elements, Cl, Ca, K and P, were in the first group, while Mn, Cu, Zn and Fe were in the second group. The factor score values for both first and second principal components for Fe were quite different in comparison with all the other elements. In the straight sixth internode (Fig. 2*b* ▶) the same groups of elements could be distinguished. However, grouping based on the factor scores was not clearly expressed. The elements within pairs K/Cl and Ca/P have similar factor score values in both twisted and straight internodes. This indicates similar distribution of these pairs of elements in both samples. However, in the twisted internode the factor scores of all four elements are alike, owing to their co-localization.

**Figure 3:**
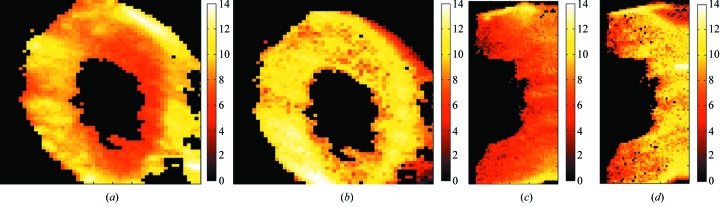
Multiplied images of element groups determined by principal component analysis, for the twisted internode (*a* and *c*) and for the straight internode (*b* and *d*). Micronutrient element (Mn, Zn, Cu, Fe) grouping is shown in (*a*) and (*b*), while the multiplied images for macronutrient elements (K, Ca, Cl, P) is presented in (*c*) and (*d*). Brighter colours on the scale bars indicate a distinct correlation while dark colours refer to a less pronounced grouping.

In order to obtain a more detailed insight of element collocations in the sections, images were multiplied element-wise according to equations (3) and (4). Fig. 3 ▶ shows image multiplications for twisted (*a*, *b*) and straight (*c*, *d*) sixth internodes. Figs. 3(*a*) ▶ and 3(*c*) represent the results of multiplication of the second group of elements (*i.e.* micronutrient elements: Cu, Zn, Mn and Fe) according to PCA (Fig. 2 ▶), while Figs. 3(*b*) and 3(*d*) ▶ show the results of multiplication of the first group of elements (macronutrient elements: P, Ca, Cl and K) for twisted and straight internodes, respectively. The spatial distribution of the elements and their collocations in the sections observed in Fig. 3 ▶ is in accordance with their grouping in Fig. 2 ▶. Elements with similar factor scores, as shown in Fig. 2 ▶, are present in the same areas of the image in Fig. 3 ▶. Such a distribution results in higher contrast of different regions of the section. This is an indication of elemental grouping in certain parts of the section. Grouping is more expressed in twisted internodes [Figs. 3(*a*) and 3(*b*) ▶] than in the corresponding straight internodes [Figs. 3(*c*) and 3(*d*) ▶]. However, correlation is more pronounced in the case of macronutrient elements (P, Ca, Cl, K) for both straight and twisted internodes [Figs. 3(*b*) and 3(*d*) ▶]. Furthermore, grouping of micronutrient elements occurs predominantly in vasculature, as well as in the sclerenchymal cell layer.

**Figure 4:**
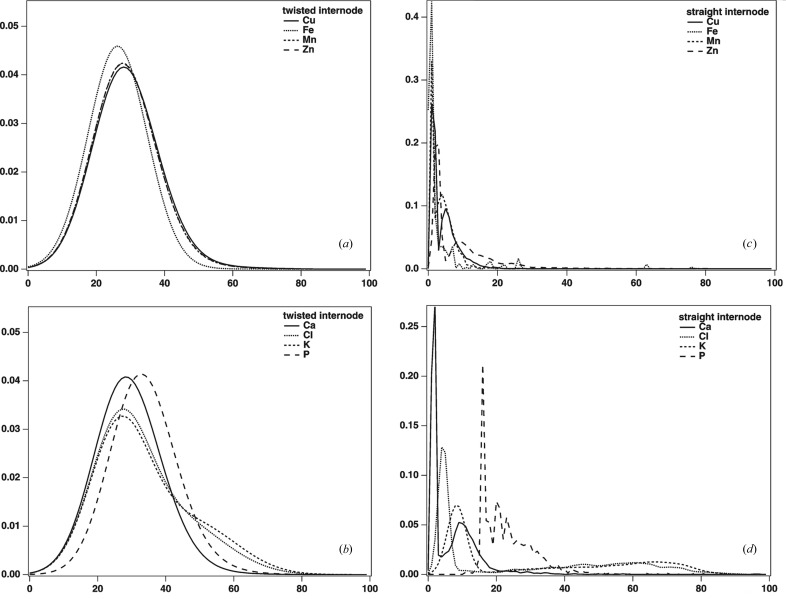
Probability density estimate plots for the individual micronutrient elements (Mn, Zn, Cu, Fe) (*a* and *c*) and macronutrient elements (K, Cl, Ca, P) (*b* and *d*) in a twisted internode and straight internode, respectively.

Fig. 4 ▶ shows PDE graphics based on X-ray microscopic images (see Fig.1 ▶). Data for the twisted internodes are displayed in Figs. 4(*a*) and 4(*b*) ▶ while the straight internodes data are presented in Figs. 4(*c*) and 4(*d*) ▶. Micronutrient element histograms are shown in Figs. 4(*a*) and 4(*c*) ▶ and macronutrient element histograms are presented in Figs. 4(*b*) and 4(*d*) ▶. The PDE analysis was in accordance with both PCA (Fig. 2 ▶) and image multiplication (Fig. 3 ▶) analyses. Similar shapes of histograms, shown in Fig. 4(*a*) ▶ for micronutrient elements in the twisted internode, correspond to similar values of factor scores in Fig. 2 ▶ as well as to distinct collocations in Fig. 3 ▶. On the other hand, different shapes of histograms, presented in Fig. 4(*c*) ▶ for micronutrient elements in the straight internode, also show less similarity by PCA and a lower correlation by image multiplication (see Fig. 3*c* ▶). In the case of macronutrient elements, differences in peak shapes and position of peak maxima are much more pronounced than for the micronutrient elements. This is particularly the case for the straight internodes.

Elemental quantification and distribution in plants are important for the understanding of plant physiology, agricultural research and food science. Various methods can be applied to determine element quantities and/or their distribution in plant cells or tissues, but almost all of these methods require special sample preparation techniques (Lombi *et al.*, 2011 ▶). Furthermore, simultaneous analysis of more than one element is often difficult and/or very time-consuming. These problems can be overcome by using µSXRF. With this imaging technique one can use freeze-dried but otherwise intact plant sections, without any additional processing or staining, while after shock-freezing and afterwards freeze-drying elemental distributions remain undisturbed. Besides simultaneous elemental mapping and quantification, µSXRF also has a very short analysis time. The latter is essential in order to avoid beam damage to the sample and allows mapping of relatively large samples. X-ray microfluorescence has often been used to study element distribution in different types of animal cells (Dučić *et al.*, 2011 ▶; Ice *et al.*, 2011 ▶), but the number of such studies in plant science is still very limited (Lombi *et al.*, 2011 ▶). This technique has also become a powerful technique for plants on the submicrometre level, and it has the potential to provide information on elemental distribution close to *in vivo* data (under cryo-conditions). Up to now, µSXRF analyses are usually used in two-dimensional raster-scanning mode with a spatial resolution in the micrometre range. For instance, to understand how plant cells segregate heavy metal Ni in the hyperaccumulator *Alyssum murale*, McNear *et al.* (2010 ▶) used µSXRF to determine the metal distribution and co-localization. Isaure *et al.* (2006 ▶) applied µSXRF to investigate Cd localization and speciation in *Arabidopsis thaliana* grown under Cd-enriched conditions. Studies of Cd compartmentalization in the hyperaccumulator *Arabidopsis halleri* implicated the mesophyll cells as sites of Cd storage (Küpper *et al.*, 2000 ▶). The distribution of Ni, Co and Zn in leaves of the Ni hyperaccumulator *Alyssum murale* was investigated by applying µSXRF imaging (Tappero *et al.*, 2007 ▶), while Young *et al.* (2006 ▶) used µSXRF to screen a large array of seeds from transgenic lines of *Arabidopsis thaliana*. Kim and co-authors visualized iron in Arabidopsis seeds, and showed that Fe is localized primarily to the provascular strands of the embryo (Kim *et al.*, 2006 ▶). This localization is completely abolished when the vacuolar iron uptake transporter VIT1 is disrupted. Iron is one of the most important micronutrients, yet problematic of the essential elements required by plants. It is needed for life-sustaining processes from photosynthesis to respiration, yet it can be toxic at high levels owing to its propensity to form hydroxyl radicals that can damage cellular constituents (Kim *et al.*, 2006 ▶). However, Fe mapping is usually difficult to distinguish since it has a distinct vascular associated distribution in plants compared with the majority of the essential nutrients. Punshon and co-authors found Fe distributed exclusively within organelles of the endodermal cells of the radicle and around the vasculature of the cotyledons of the Arabidopsis seeds embryo, although Zn was distributed uniformly throughout the cells, strongly co-localizing with Ca (Punshon *et al.*, 2009 ▶), similar to our µSXRF results in *D. balcanica* stem (Fig. 1 ▶).

Usually µSXRF analyses have been performed mostly on leaves and roots and this technique had not yet been applied to analyse sectioned plants stems. In our experiments we analysed the elemental distribution in the stem sections from straight and twisted internodes of *D. balcanica*. We found that elemental accumulation and grouping are different in the corresponding segments of straight and twisted internodes. Climbing plants have evolved various mechanisms to climb and fix their stem around solid supports (Carlquist, 1985 ▶). Liana plants exhibit thigmotropic response, induced by a contact with mechanical structure, resulting in twining and fixing of the stem around solid support (Telewski, 2006 ▶). Thigmomechanoperception occurs in the apical region, while the signal is rapidly acropetaly transmitted to the actively elongating zone directly below the apical meristem. Physiological response to mechanical signal includes a cascade of processes (*i.e.* change in action potentials, increase in intracellular Ca^2+^, H_2_O_2_ and reactive oxygen species levels, expression of calmoduline an calmodulin related genes, ethylene biosynthesis) which all returns to premechanical signal levels in 24 h (Telewski, 2006 ▶). Twining stems elongate maximally before coiling (Meloche *et al.*, 2007 ▶).

Herein, we have demonstrated that combining µSXRF analysis with advanced statistical and image analysis methods has enabled us to study elemental distribution and co-localization in the stem sections of twisted and straight sixth internodes of monocotile liana plants. The results show that macronutrient elements (K, Ca, Cl and P) are distributed homogeneously in both straight and twisted internodes. In contrast, micronutrient elements (Cu, Zn Mn and Fe) are mostly localized in the vasculature, as well as in the layer of sclerenchyma cells (Figs. 1 ▶ and 3 ▶). The accumulation and co-localization of different elements is more expressed in twisted internodes. These differences may possibly be related to the structural differences between straight and twisted internodes: twisted internodes are characterized by the presence of gelatinous fibers in the sclerenchyma cell layer and in vascular bundles, while gelatinous fibers are rare or absent from regions of stem that do not twine (Bowling & Vaughn, 2009 ▶). Increased lignification characterizes parts of the liana stem with strong twining force (Köhler *et al.*, 2000 ▶), spatially expressed in the region of contact with the support (Meloche *et al.*, 2007 ▶). Correlation between increased lignification in twined stem sections and asymmetric element distribution indicates that the peculiar elemental co-localization occurs at the side of the segment of twisted internode that was in contact with the support (Meloche *et al.*, 2007 ▶). It is known that some anatomical adaptations to mechanical strain in lianas involve high flexibility of the tissue structures. Such adaptations may lead to elemental redistribution in the twisted parts of the stem, and also a different distribution pattern for different groups of elements. A suitable combination of metabolite and micronutrient element analysis is necessary to determine possible regulation of the metabolic reactions just before a prevalent synthesis of pharmaceutically important metabolite such as, for example, diosgenin.

Here the presented different image analyses are useful for studies of plant elemental distribution related to plant reactions to environmental or metabolic changes. It was already observed that metal stress enhances diosgenin yield in *Dioscorea bulbifera* L. cultures (Narula *et al.*, 2005 ▶). On the other hand our results show application of statistical and image analyses as a tool for better interpretation of results obtained by µSXRF. In the *D. balcanica* model system the collocation is generally less distinct in the case of macronutrient elements in both straight and twisted internodes. Distribution of the individual elements in the sections (Fig. 4 ▶) is in accordance with their group distributions and co-localization (Figs. 2 ▶ and 3 ▶). In view of the fact that different methods used in this study confirmed the same results, this shows the reliability and accuracy of the methods used. Constitutive elements such as Ca, K, Cl and P are present in every living cell, thus it is expected that their distribution is less localized in different regions of plant tissues. Multiplied images for these macronutrient elements (Fig. 3 ▶) show less contrast compared with multiplied images for the micronutrient elements. This result is supported by the more complex and also highly variable PDE histograms of macronutrient elements compared with those of the micronutrient elements. PDE histograms of micronutrient elements have similar shapes in terms of maximum position and intensity and thus reflect a distinct and correlated element distribution (grouping).

This study illustrates the potential of the µSXRF imaging data processed with the multivariate image analyses which may considerably improve the results interpretation. Nevertheless, the results shown here concerning the physiological processes after thygmotropic response and different distribution of elements in straight and twisted internodes must be considered with caution. Because of the experimental set-up, a relatively large straight internode was not completely imaged and possible differences in the entire cross section of the stem sample cannot be excluded. Nevertheless, the multivariate imaging approach presented here may be used in future µSXRF studies of various biological samples.

## 4. Conclusion

The data presented here could not be obtained by techniques that do not map elemental distribution in two-dimensions, arguing again in favour of µSXRF over many other analytical approaches. Interestingly, the analysis approaches we used segregated micronutrient elements from macronutrient elements. In our approach, statistical methods and image analysis have been used to extract more information from µSXRF data, as well as to create elemental co-distribution images with a more pronounced contrast. Here we show an example of the quantity, distribution and grouping/co-localization of various elements in stems of the monocotyledonous climber *D. balcanica*. Macronutrient elements (K, P, Ca, Cl) are distributed homogeneously in both straight and twisted internodes. Micronutrient elements such as Mn, Fe and Cu predominantly group in the vasculature and in the sclerenchyma cell layer.

## References

[bb1] Bowling, A. J. & Vaughn, K. C. (2009). *Am. J. Bot.* **96**, 719–727. 10.3732/ajb.0800373 21628227

[bb2] Carlquist, S. (1985). *Aliso*, **11**, 139–157.

[bb3] Carlquist, S. (1991). *The Biology of Vines*, edited by F. E. Putz and H. A. Mooney, pp. 53–71. Cambridge University Press.

[bb6] Dučić, T., Quintes, S., Nave, K. A., Susini, J., Rak, M., Tucoulou, R., Alevra, M., Guttmann, P. & Salditt, T. (2011). *J. Struct. Biol.* **173**, 202–212. 10.1016/j.jsb.2010.10.001 20950687

[bb7] Furmanowa, M. & Guzewska, J. (1988). *Biotechnology in Agriculture and Forestry 7, Medicinal and Aromatic Plants II*, edited by Y. P. S. Bajaj, pp. 162–184. Berlin: Springer.

[bb8] Hinton, R. W. (2007). *Geostand. Newsl.* **23**, 197–207.

[bb9] Ice, G. E., Budai, J. D. & Pang, J. W. (2011). *Science*, **334**, 1234–1239. 10.1126/science.1202366 22144618

[bb10] Isaure, M. P., Fayard, B., Sarret, G., Pairis, S. & Bourguignon, J. (2006). *Spectrochim. Acta B*, **61**, 1242–1252.

[bb11] Isnard, S., Cobb, A. R., Holbrook, N. M., Zwieniecki, M. & Dumais, J. (2009). *Proc. R. Soc. B*, **276**, 2643–2650. 10.1098/rspb.2009.0380 PMC2686668 19386656

[bb12] Kim, S. A., Punshon, T., Lanzirotti, A., Li, L., Alonso, J. M., Ecker, J. R., Kaplan, J. & Guerinot, M. L. (2006). *Science*, **314**, 1295–1298. 10.1126/science.1132563 17082420

[bb13] Köhler, L., Speck, T. & Spatz, H. C. (2000). *Planta*, **210**, 691–700. 10.1007/s004250050669 10805439

[bb14] Küpper, H., Lombi, E., Zhao, F. J. & McGrath, S. P. (2000). *Planta*, **212**, 75–84. 10.1007/s004250000366 11219586

[bb15] Lombi, E., Scheckel, K. G. & Kempson, I. M. (2011). *Environ. Exp. Bot.* **72**, 3–17.

[bb16] McNear, D. H., Chaney, R. L. & Sparks, D. L. (2010). *Phytochemistry*, **71**, 188–200. 10.1016/j.phytochem.2009.10.023 19954803

[bb18] Meloche, C. G., Knox, J. P. & Vaughn, K. C. (2007). *Planta*, **225**, 485–498. 10.1007/s00425-006-0363-4 16955273

[bb19] Narula, A., Kumar, S. & Srivastava, P. S. (2005). *Plant Cell Rep.* **24**, 250–254. 10.1007/s00299-005-0945-9 15809888

[bb20] Paunesku, T., Vogt, S., Irving, T. C., Lai, B., Barrea, R. A., Maser, J. & Woloschak, G. E. (2009). *Int. J. Radiat. Biol.* **85**, 710–713. 10.1080/09553000903009514 19637082

[bb21] Punshon, T., Guerinot, M. L. & Lanzirotti, A. (2009). *Ann. Bot.* **103**, 665–672. 10.1093/aob/mcn264 PMC2707871 19182222

[bb22] Sakdinawat, A. & Attwood, D. (2010). *Nat. Photon.* **4**, 840–848.

[bb23] Šavikin-Fodulović, K., Grubišić, D., Ćulafić, L., Menković, N. & Ristić, M. (1998). *Plant Sci.* **135**, 63–67.

[bb24] Solé, V. A., Papillon, E., Cotte, M., Walter, P. & Susini, J. (2007). *Spectrochim. Acta B*, **62**, 63–68.

[bb25] Tappero, R., Peltier, E., Gräfe, M., Heidel, K., Ginder-Vogel, M., Livi, K. J., Rivers, M. L., Marcus, M. A., Chaney, R. L. & Sparks, D. L. (2007). *New Phytol.* **175**, 641–654. 10.1111/j.1469-8137.2007.02134.x 17688581

[bb26] Telewski, F. W. (2006). *Am. J. Bot.* **93**, 1466–1476. 10.3732/ajb.93.10.1466 21642094

[bb27] Vekemans, B., Janssens, K., Vincze, L., Adams, F. & Van Espen, P. (1995). *Spectrochim. Acta B*, **50**, 149–169.

[bb28] Vekemans, B., Janssens, K., Vincze, L., Adams, F. & Van Espen, P. (2004). *X-ray Spectrom.* **23**, 278–285.

[bb29] Young, L. W., Westcott, N. D., Attenkofer, K. & Reaney, M. J. T. (2006). *J. Synchrotron Rad.* **13**, 304–313. 10.1107/S0909049506019571 16799221

